# A New Method for the Sputum Cytology Test Without Direct Contact to Specimens During COVID-19 Pandemic

**DOI:** 10.3389/fmed.2021.746731

**Published:** 2022-01-28

**Authors:** Junqi Cui, Xia Wang, Yamin Rao, Tianhai Ji, Long Li

**Affiliations:** ^1^Department of Pathology, Shanghai Ninth People's Hospital, Shanghai Jiaotong University School of Medicine, Shanghai, China; ^2^Department of Cardiology, Shanghai Chest Hospital, Shanghai Jiaotong University, Shanghai, China; ^3^Department of Urology, Shanghai Ninth People's Hospital, Shanghai Jiaotong University School of Medicine, Shanghai, China

**Keywords:** COVID-19, sputum cytology, sealed bag, protection, medical alcohol

## Abstract

**Background:**

Coronavirus disease 2019 (COVID-19) pandemic continues to spread across the world. Specimens of blood, body fluids and excreta received in the department of pathology undoubtedly increased the risk of infection, especially in some hospitals that are short of professional protection capability. Here we provided a new simple way for the sputum cytology test during the COVID-19 pandemic.

**Methods:**

Sputum samples from 30 patients with lung cancer were collected and divided into two groups, including the control group and the experimental group. Samples of the control group were processed in the biological safety cabinet, while the experimental group was put into the sealed specimen bag directly and pretreated with 75% medical alcohol. Then the cell morphology and tumor cell identification were analyzed by cell smears and liquid-based cell staining. The expression of cell antigens was determined by immunohistochemical staining.

**Result:**

Our result showed that both sputum samples in two groups exhibited complete cell structure and clear morphology according to the cell smear and liquid-based cell staining. In addition, the immunohistochemical result showed that cell antigens, including cytokeratin (CK), leukocyte common antigen (LCA), and thyroid transcription factor-1 (TTF1), were specifically expressed in the cell membrane, cytoplasm, and nucleus, respectively. The tumor cells were distributed diffusely, and cell antigens were located accurately after pretreatment with 75% medical alcohol and were consistent with that of the control group.

**Conclusion:**

Using 75% medical alcohol to pretreat sputum specimens has no obvious impact on cell morphology and antigens expression. Our study provided a new method for the sputum cytology test with no direct contact so as to protect medical staff against the virus during COVID-19 outbreak.

## Background

Coronavirus disease 2019 (COVID-19) was first detected in China in December 2019, and declared as a pandemic by the WHO on March 11, 2020 (1–3). During the period of COVID-19, the specimens of blood, body fluids, and excreta received in the department of pathology increased the risk of infection (4, 5). Pathology technicians in the middle- and small-scale hospitals, which are short of professional protection capabilities, such as negative pressure laboratories and microbiology laboratories, are more susceptible to virus infection, as they are often exposed to a variety of pathological samples of patients (6, 7). Besides, as the specimen collected for clinical examination is of great uncertainty, it is easy for medical staff to be infected by sputtering or aerosol after opening the sample boxes directly (8). In some backward areas, medical staff cannot be evenly equipped with N95 masks, goggles, and protective clothing. Therefore, an efficient way is needed for the test of pathological specimens during the pandemic (9). Here, we proposed a simple and feasible way to pretreat sputum samples by contactless disinfection, and cell smears, liquid-based cell staining, and immunohistochemical staining were performed to investigate whether this new method could affect the pathological observation and cell antigen expression in the samples of patients with lung cancer (10).

## Materials and Methods

### Material Preparation

**Figure 1:**
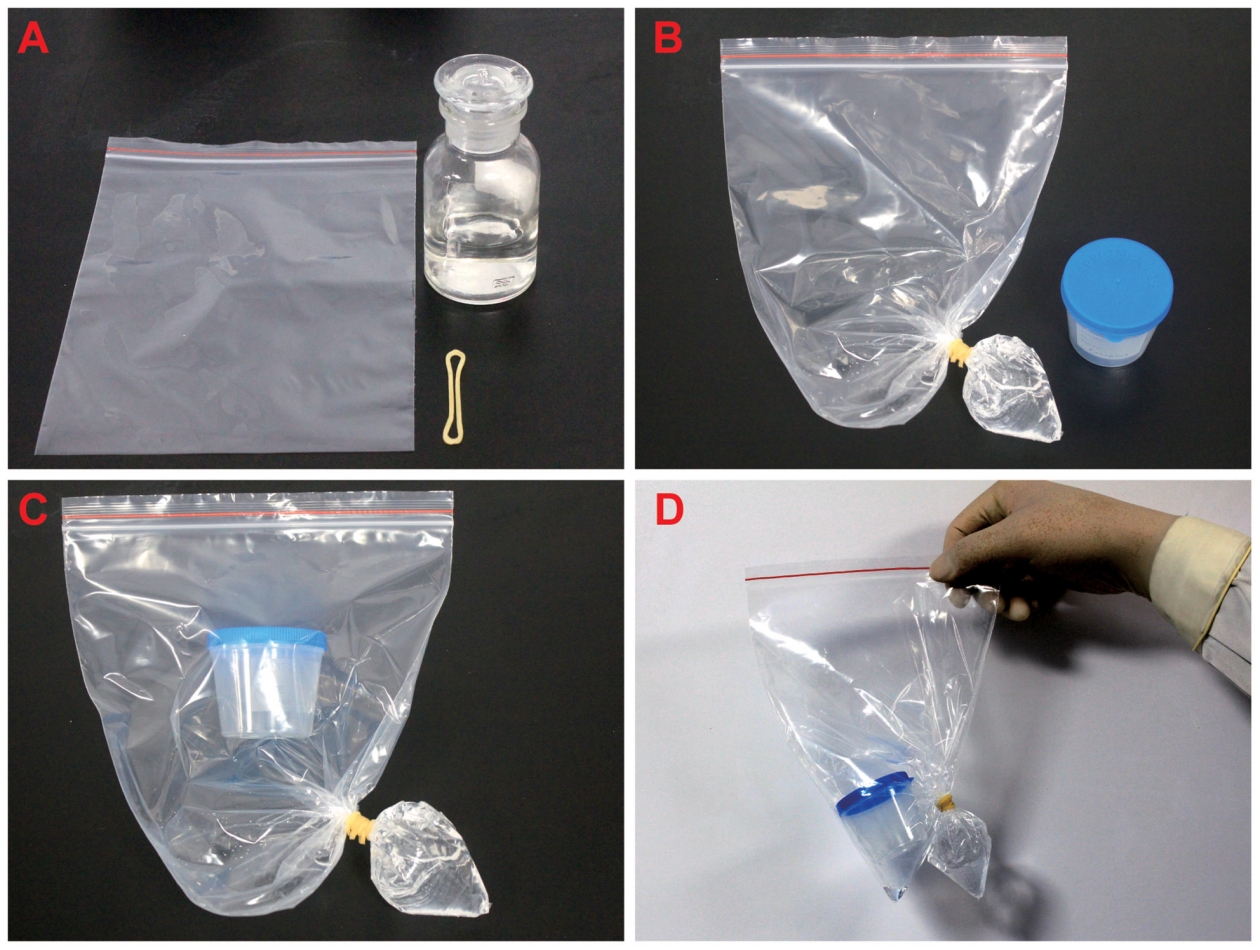
The processing of pathological samples. **(A)** Prepare 75% alcohol, rubber bands, and seal bag before the experiment. **(B)** The alcohol was injected into the sealed bag and the corner of the bag was fastened with a rubber band and the bag was sealed. **(C,D)** The sample box containing sputum was transferred to the sealed bag, and then the specimen bag was sealed.

The experimental materials are the sealed specimen bag, rubber band, and 75% medical alcohol. Initially, 30 ml of alcohol was injected into the sealed bag and the sealed bag was tilted so that the alcohol can flow to the corner of one side of the bag. Then the corner of the bag was fastened with a rubber band (Figures 1A,B).

### Specimen Collection

Sputum samples were collected from 30 patients with lung cancer in the respiratory department of the Ninth People's Hospital affiliated with the Shanghai Jiaotong University, School of Medicine from February to July in 2020. Two copies of each sample were collected from one patient, one of the samples act as the control group and another was the experimental group. Samples of the control group were placed into a biosafety box and sent to the laboratory. All the next steps were performed in the biosafety cabinet. Samples of the experimental group were directly put into a sealed specimen bag and treated with 75% medical alcohol, then were processed to the next step the same as the control group.

### Cytological Specimen Preparation

**Figure 2:**
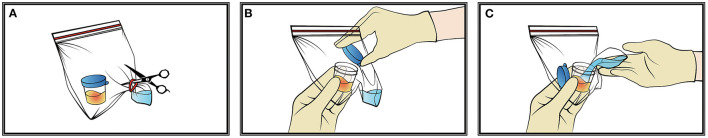
The pretreatment of pathological samples. **(A–C)** The specimen box was opened inside the sealed bag, then cut the rubber band and squeezed the bottom of the specimen bag so that the alcohol could flow into the specimen box.

Samples of the control group were conducted according to the routine cytology experiment procedure. All the processes were performed in the biosafety cabinet for cytology staining and immunohistochemistry. The process of the experimental group was done as follows:

1. Sputum samples were placed into a specimen box and transferred to the specimen bag. After adding 75% of medical alcohol, the corner of the bag was fastened with a rubber band and the specimen bag was sealed (Figures 1B–D).
2. The specimen box was opened inside the sealed bag. Then to cut the rubber band and squeeze the bottom of the specimen bag, so that the alcohol could flow into the specimen box. Close the lid of the specimen box and keep it stand for 20 min so that the virus and bacteria can be inactivated by the alcohol (Figure 2). A total of 75% of medical alcohol can be used for tissue fixation of alveolar lavage fluid (11). After that, the cytology staining and immunohistochemistry were prepared according to the routine method.

### Cytological Staining

Cytological staining was done as previously reported. Briefly, cytological specimens were placed in a 15 ml centrifuge tube and centrifuged at 2,500 rpm for 5 min. After that, part of the precipitate was collected to perform a cell smear, while the rest was collected for liquid-based staining by using a liquid-based cell preservation solution.

### Immunohistochemistry

After centrifugation, samples were wrapped with sponge paper and put into a dehydrator for dehydration. After dehydration, samples were soaked into the wax and cut into slices. Then slices were baked for 1 h and repaired with pH 9.0 ethylenediaminetetraacetic acid (EDTA) for 20 min. After that slices were incubated with primary antibody against CK (1:100, Cell Signaling Technology, MA, USA), LCA) (1:100, Cell Signaling Technology, MA, USA) orTTF1 (1:100, Cell Signaling Technology, MA, USA) at room temperature for 1 h. The color reaction was then made with DAB staining.

### Liquid Waste Disposal

The used specimen box was put into a sealed bag and re-sealed. Then it was disposed of as medical waste.

## Results

### Pretreatment With Alcohol Had no Significant Impact on the Observation of Cell Structure and Morphology

**Figure 3:**
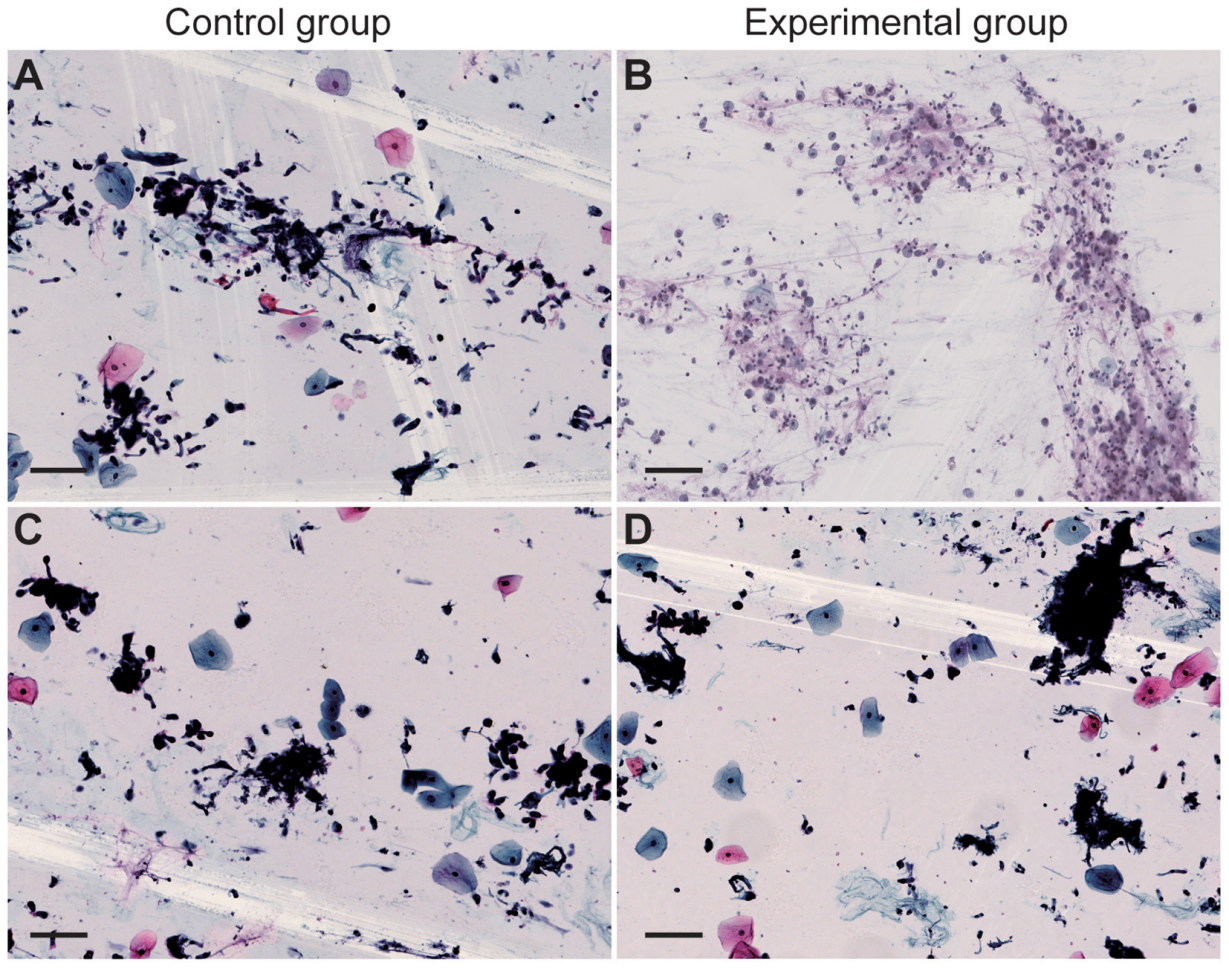
Cell smear and liquid-based cell staining of sputum were performed. Cytological specimens were placed in a 15-ml centrifuge tube and centrifuged at 2,500 rpm for 5 min. **(A,B)** Cell smears of the control group and the experimental group, respectively. **(C,D)** Liquid-based cell staining of the control group and the experimental group, respectively. Scale bar = 100 μm.

The cell smears and liquid-based cell staining results showed that the structure of tumor cells and other kinds of cells in two groups are intact with clear nuclei and a clean background (Figure 3). The number and morphology of cells fit the requirements of reading, suggesting that the quality of cell slides made from disinfected cells treated with 75% medical alcohol was similar to that of the control group. Our result demonstrated that disinfection by 75% medical alcohol had no significant impact on the cell structure and morphology and interpretation of pathological results.

### Pretreatment With Alcohol Had no Significant Impact on the Expression of Cell Antigens

**Figure 4:**
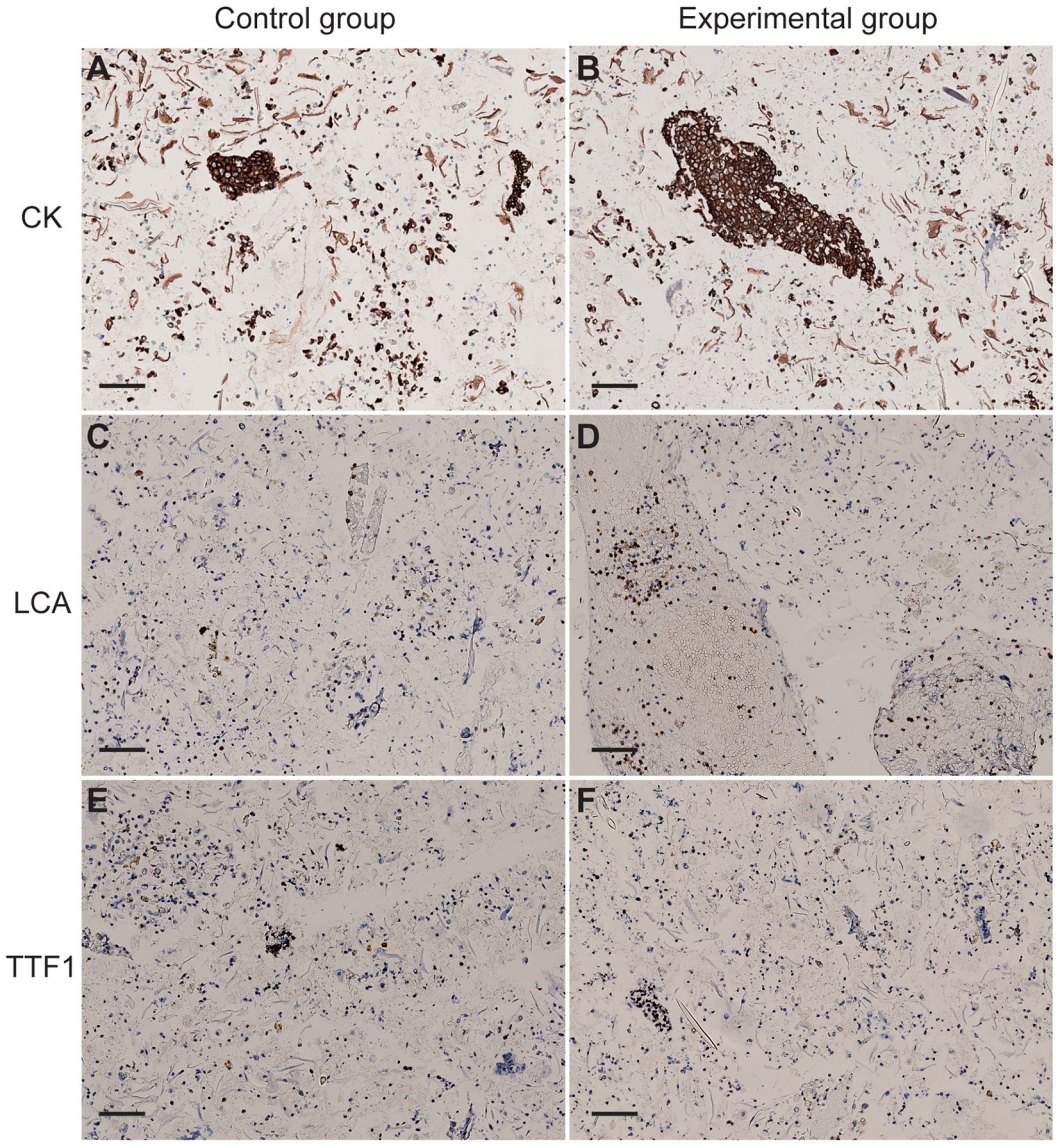
The expression of cytokeratin (CK), leukocyte common antigen (LCA), and thyroid transcription factor-1 (TTF1) and in cells of sputum. **(A,B)** CK was expressed on the membrane of cells in the control group and the experimental group. **(C,D)** LCA was expressed in the cytoplasm of cells in the control group and the experimental group. **(E,F)** TTF1 was expressed in the nucleus of cells in the control group and the experimental group. Scale bar = 100 μm.

Immunohistochemical results showed that the expression of cell antigens is complete, and the localization of cell antigens is clear and accurate. Cell antigens, such as CK and LCA, was expressed on the membrane and cytoplasm, respectively (Figures 4A–D), and TTF1 was located in the nucleus (Figures 4E,F), suggesting that 75% of medical alcohol pretreatment for sputum did not affect the expression pattern of cell antigens.

## Discussion

In January 2020, China reported the outbreak of severe acute respiratory syndrome coronavirus 2 (SARS-CoV-2) that was further declared as a pandemic owing to rapid spread to different parts of the world (12). Although COVID-19 is a contagious disease that spreads rapidly *via* droplet particles arising through sneezing and coughing action of an infected person, COVID-19 Diagnosis and Treatment Protocol (Trial version 6), released by the National Health Commission (NHC) on 19 February 2020 pointed out the possibility of virus transmission through aerosols when prolonged exposure to high concentrations of aerosols in a relatively closed environment (13).

During COVID-19 pandemic, pathological specimens, such as sputum and lavage fluid, from the department of respiratory and emergency may be at risk of infecting medical staff. Thus, sputtering and aerosol transmission are very likely to occur if specimens are processed routinely. At present, pathology departments in some middle- and small-scale hospitals are still suffering from a lack of a simple and efficient way to process the cytological specimen without destruction of cell morphology. What is more, some middle- and small-scale hospitals were not even equipped with professional negative pressure laboratories and biosafety cabinets, which may cause cross-infection among medical staff during the process of handling pathological specimens. In addition, it is very likely to trigger contact infection by aerosol if specimen boxes containing samples are opened directly.

As the cytological examination of sputum and lavage fluid is the major way to diagnose respiratory diseases, we first propose a new and economic way to process the specimens efficiently. In this study, we used a sealed bag to keep the sputum sample in a closed space, and then pretreated it with 75% medical alcohol to sterilize the sample, to protect the technician and other staff against the virus. The sealed pathological specimen bag has good corrosion resistance and fastness, and is not easy to be damaged during operation. The rubber band was used to prevent the alcohol from shaking in the bag. The seal strip was used to isolate the virus that may exist after the sample box was opened. As the 75% medical alcohol can cause protein coagulation, which may affect the sputum cytology test. In this study, we verified that the cell morphology and cell antigens expression are not affected after medical alcohol pretreatment by using cell smears, liquid-based cell staining, and immunohistochemical staining. Thus we conclude that this method has the advantage of being low cost and easy to operate, it is worth popularizing and applying in pathology departments, especially hospitals in backward areas.

## Conclusion

This study provided a new method for technicians to test samples that may contain pathogenic bacteria or viruses without direct contact to protect medical staff against viruses during COVID-19 outbreak.

## Data Availability Statement

The original contributions presented in the study are included in the article/supplementary material, further inquiries can be directed to the corresponding author/s.

## Ethics Statement

The studies involving human participants were reviewed and approved by ethics committee on human research at Shanghai Jiaotong University, School of Medicine, Shanghai Ninth People's Hospital. The patients/participants provided written informed consent to participate in this study.

## Author Contributions

JC and XW wrote the manuscript and contributed equally to this work. LL, TJ, and YR conduct experimental research, organize experimental data into manuscripts, and supervised the findings of this work. All authors contributed to the article and approved the submitted version.

## Funding

This study was supported by the Sponsored by Shanghai Sailing Program (No. 19YF1427600) and the National Nature Science Foundation of China (No. 81900438).

## Conflict of Interest

The authors declare that the research was conducted in the absence of any commercial or financial relationships that could be construed as a potential conflict of interest.

## Publisher's Note

All claims expressed in this article are solely those of the authors and do not necessarily represent those of their affiliated organizations, or those of the publisher, the editors and the reviewers. Any product that may be evaluated in this article, or claim that may be made by its manufacturer, is not guaranteed or endorsed by the publisher.
